# Influence of Chemical, Morphological, Spectroscopic and Calorimetric Properties of Agroindustrial Cellulose Wastes on Drainage Behavior in Stone Mastic Asphalt Mixtures

**DOI:** 10.3390/ma17215278

**Published:** 2024-10-30

**Authors:** Laura Yessenia Cabello-Suárez, José Anzaldo Hernández, José Roberto Galaviz-González, David Avalos-Cueva, Edgar Benjamín Figueroa Ochoa, Daniel Escobar Hernández, Manuel Alberto Gallardo-Sánchez, Pedro Limón-Covarrubias, Emma Rebeca Macías-Balleza

**Affiliations:** 1 Department of Project Engineering, Universidad de Guadalajara, 48 Blvd. José Guadalupe Zuno, Zapopan C.P. 45157, Jal., Mexico; laura.cabello8788@alumnos.udg.mx; 2 Department of Wood, Cellulose and Paper, Universidad de Guadalajara, 440 Cam. Ramón Padilla Sánchez, Las Agujas C.P. 45221, Jal., Mexico; j.anzaldo@academicos.udg.mx; 3 Department of Civil Engineering and Topography, Universidad de Guadalajara, 1421 Blvd. Marcelino García Barragán, Guadalajara C.P. 44430, Jal., Mexico; jose.galaviz2401@academicos.udg.mx (J.R.G.-G.); david.avalos@academicos.udg.mx (D.A.-C.); manuel.gallardo@academicos.udg.mx (M.A.G.-S.); pedro.limon@academicos.udg.mx (P.L.-C.); 4 Department of Chemistry, Universidad de Guadalajara, 1421 Blvd. Marcelino García Barragán, Guadalajara C.P. 44430, Jal., Mexico; benjamin.figueroa@academicos.udg.mx (E.B.F.O.); daniel.ehernandez@academicos.udg.mx (D.E.H.); 5 Department of Chemical Engineering, Universidad de Guadalajara, 1421 Blvd. Marcelino García Barragán, Guadalajara C.P. 44430, Jal., Mexico

**Keywords:** asphalt drainage, asphalt mixture, cellulose pulp, lignocellulosic

## Abstract

New asphalt mixtures have been improved by using fibers (polypropylene, polyester, asbestos, carbon, glass, nylon, lignin, coconut, sisal, recycled rubber, PET, wood, bamboo, and cellulose), reducing the temperature and compaction energy for their collocation, minimizing the impact on the environment, increasing the tenacity and resistance to cracking of hot mix asphalt (HMA), preventing asphalt drainage in a Stone Mastic Asphalt (SMA). Hence, this paper aims to evaluate the influence of the chemical (lignin content, ash, viscosity, degree of polymerization, and elemental analysis), morphological (SEM), spectroscopic (FTIR-ATR and XRD), and calorimetric (ATG and DSC) properties of celluloses from bagasse *Agave tequilana* Weber var. Azul (ABP), corrugated paperboard (CPB) and commercial cellulose fiber (CC) as Schellenberg drainage (D) inhibitors of the SMA. The ABP was obtained through a chemical process by alkaline cooking, while CPB by a mechanical refining process. The chemical, morphological, spectroscopic, and calorimetric properties were similar among the analyzed celluloses, but CPB and ABP cellulose are excellent alternatives to CC cellulose for inhibiting drainage. However, CPB is the most effective at low concentrations. This is attributed to its morphology, which includes roughness, waviness, filament length, orientation, and diameter, as well as its lignin content and crystallinity.

## 1. Introduction

The rapid development of urbanization, along with the increase in traffic load, volume, and speed, has negatively impacted the ecosystem through energy consumption, CO_2_ emissions, water discharges, and solid waste generation in pavement manufacturing [1,2]. Flexible pavements made with asphalt mix are of particular interest because they deteriorate under loading as they age, causing the wearing surface to become uneven for traffic [3]. Conventional mixes, such as hot mix asphalt (HMA), are very sensitive to several factors, including temperature, load level, load duration, and humidity [4]. Thus, the use of discontinuous granulometry asphalt mixes, such as Stone Mastic Asphalt (SMA), is becoming increasingly common, as they demonstrate good resistance to permanent deformation, wear, and the appearance of premature fatigue cracks compared to hot mix asphalt (HMA) [5]. Its texture provides greater contact adherence between the tire and the pavement, enhancing safety for the user [6]. SMA mixes typically have a coarser aggregate particle size composition and a higher asphalt content than HMA mixes, which necessitates the use of stabilizing fibers to prevent asphalt drainage [5,7,8]. Among the most commonly used fibers are lignocellulose fibers.

Lignocellulose is a naturally occurring material with a complex structure consisting of three main biopolymers: cellulose (a glucose polymer), hemicellulose (a polymer primarily composed of pentoses), and lignin (a phenolic polymer) [9,10]. The structure and composition of these biopolymers vary greatly depending on several parameters related to their source, such as the type of plant species and its growing conditions [9].

The most common lignocellulose fibers are derived from waste paper and are added to the SMA asphalt mix as a stabilizer at concentrations between 0.3% and 0.5% [7], either fluffed or in pellets [4]. These fibers inhibit asphalt drainage in the mix, promoting better distribution of the asphalt and forming a thick, homogeneous film around the aggregates [8]. However, these fibers are not produced domestically, leading to an import process that increases the final cost of the mix compared to HMA [5,11]. While the cellulose fiber currently in use improves the drainage of the mixture, it has limitations, particularly in terms of fatigue and moisture resistance [4], and poses a critical issue in rutting resistance. Therefore, it is important to emphasize the selection of fibers or fiber combinations to optimize the performance of both the asphalt and the asphalt mixture. Considering this, De la Cruz Analya [8] demonstrated that for an optimum asphalt content of 6.52%, a percentage of 0.40% Sudaglass basalt fibers (L = 6 mm, thickness = 13 µm) was necessary, resulting in an asphalt drainage value of 0.10%. The study concluded that basalt fibers could substitute conventional cellulose fibers to stabilize the SMA mixture. Unfortunately, the cost of basalt fiber was found to be higher than that of cellulose. Huang et al. [4] aimed to achieve a better cost-benefit ratio and discovered that adding 0.40% cellulose-basalt fiber in a 50/50 ratio resulted in favorable drainage behavior of the asphalt mixture, with an asphalt drainage value of 0.125%. They concluded that hybrid modification could provide a more balanced cost-benefit performance. On the other hand, Preciado Bolívar and Sierra Martínez [11] compared two SMA mixtures: one containing 0.3% pelletized cellulose fiber and the other made with coconut husk fiber. The SMAs were tested for asphalt drainage, revealing that the addition of 0.05% coconut fiber inhibited asphalt drainage in the mix. Similarly, Oda et al. [5] provided a comparison in which they used natural coconut and sisal fibers alongside paper cellulose fibers added at 0.3% and 0.5% in an SMA mix with 6.8% asphalt content. They found that using coconut and sisal fibers at 0.5% resulted in asphalt drainage similar to that observed with paper cellulose fiber.

At the same time, vegetable fibers play an important role in SMA mixtures, as their use reduces contamination generated by these materials and is environmentally friendly [12]. Additionally, these fibers possess excellent mechanical and microstructural properties; they are biodegradable, non-abrasive, eco-adaptable, non-toxic, economical, and accessible [13,14]. They also have low density and a good strength-to-weight ratio, which means that the smaller the fibers are, the lighter and stronger they can potentially become [15,16]. Furthermore, the higher the cellulose content and crystallinity, the more resistant properties they will impart to the material to which they are added [14]. The main component of natural fibers such as cotton, flax, hemp, jute, sisal, and agave is cellulose, which provides the fiber’s mechanical properties. This cellulose is arranged in microfibrils, surrounded by two primary components: lignin and hemicelluloses [17]. The cellulose content of natural fibers typically ranges from 60% to 80%, while lignin content ranges from 5% to 20%. The remainder consists of hemicellulose, pectin, water, and low molecular-weight sugars [18]. Cellulose can be obtained through chemical or mechanical processes. The chemical process involves cooking wood or natural fibers in a soda, sulfite, and Kraft solution at high temperatures and pressures. This method dissolves varying degrees of lignin in the lignocellulosic material, releasing the fibrils with yields ranging from 35% to 50% [19]. These performances are influenced by several factors, including species, cooking reagent concentration, digestion time, and temperature. Pulps treated with alkaline soda cooking are generally easier to bleach and are less likely to lose their properties over time. Gallardo-Sánchez et al. [20] report the production of cellulose pulp from *Agave tequilana* bagasse by boiling it in a 20% sodium hydroxide (NaOH) solution with 0.1% anthraquinone and a hydromodule of 5:1 at a temperature of 170 °C for 90 min, yielding 40% cellulose pulp. The mechanical process involves subjecting mechanically ground wood or natural fibers to high temperatures and pressures, achieving yields of 85–95%. However, much of the lignin remains in the pulp, and this mechanical processing results in significantly shorter and more fragmented fibers [21]. It is important to note that cellulose nanofibrils and nanocrystals possess excellent properties, including a high specific surface area, good mechanical properties, light weight, and non-toxicity. However, the high production costs associated with these materials hinder their application in large volumes [22,23,24], making their use in asphalt mixtures impractical.

The bagasse of *Agave tequilana* Weber Var. Azul (ABF) is a residue generated from the production of tequila from the agave plant. Tequila is an internationally consumed liquor with a designation of origin, cultivated in a limited and exclusive manner in 181 municipalities across five Mexican states: Jalisco (125 municipalities), Michoacán (30), Tamaulipas (11), Nayarit (8), and Guanajuato (7) [25]. ABF is a lignocellulosic material composed mainly of cellulose (70% to 80%), lignin (15% to 20%), and hemicellulose (5% to 10%) [20]. According to the Consejo Regulador del Tequila [26], between 2017 and 2023, an average of 1,680,286 tons of tequila agave were processed, with waste accounting for 40% of the total weight of the agave [27], resulting in an average ABF waste of 672,114 tons. This waste has been utilized to produce alcohols, organic acids, compost, paper, fuel, edible mushrooms, and animal feed. However, the generation rate of this waste exceeds its utilization [27,28,29]. Another lignocellulosic waste available on a massive scale is paperboard waste, which is also prone to recycling. However, these processes cannot be repeated indefinitely, as each cycle causes the fibers to lose strength, affecting the quality of the recycled paper. Consequently, the material often does not meet quality standards prior to the manufacturing process and is discarded.

The literature indicates that population growth and increased traffic have generated negative environmental impacts on pavement manufacturing. Conventional asphalt mixes (HMA) have been particularly affected due to their sensitivity to factors such as increased loads, temperature, and humidity. Consequently, the use of Stone Mastic Asphalt (SMA) mixes has been promoted, as they offer better resistance and safety. However, SMA mixes require a significant amount of asphalt, which can lead to drainage issues. To stabilize the asphalt and reduce drainage, lignocellulosic fibers are incorporated, thereby improving durability. Therefore, utilizing natural fibers from agroindustrial waste, such as agave bagasse and paperboard, can help lower the cost of SMA mixes while also minimizing their harmful environmental impact.

Magaña-Orozco [30] evaluated the performance of adding *Agave tequilana* bagasse to an SMA blend by assessing volumetric properties, drainage, moisture susceptibility, and resistance to permanent deformation, comparing its behavior against commercial cellulose. The study concluded that with 1% bagasse, the standardized volumetric requirements were met, and moisture susceptibility was not significantly affected by the type of fiber, while permanent deformation was reduced compared to commercial fiber. However, this percentage of bagasse is significantly higher than the amount required by the AASHTO M325 standard [7] or cellulose fiber usage. Natural fibers are hydrophilic, and when combined with hydrophobic matrices, poor adhesion can occur between the fiber and the matrix, negatively affecting mechanical behavior and performance [31]. Additionally, Huang et al. [4] highlighted that higher cellulose content increases asphalt content, leading to elevated costs. Moreover, the increased asphalt content can affect performance, reducing resistance to permanent deformation and accelerating aging during the service life of the mixture [32].

Therefore, further studies are needed to develop optimized cellulose formulations and their appropriate applications to enhance both the performance and cost-effectiveness of SMA asphalt mixtures. Consequently, the objective of this work is to evaluate the influence of the chemical properties (lignin and ash content, viscosity, degree of polymerization, and elemental analysis), morphological properties (SEM), spectroscopic properties (FTIR-ATR and XRD), and calorimetric properties (ATG and DSC) of celluloses derived from *Agave tequilana* bagasse (ABP), corrugated paperboard (CPB), and commercial cellulose (CC) as Schellenberg drainage (D) inhibitors in the SMA asphalt mixture. This evaluation aims to utilize these materials in developing value-added products and to minimize the pollution generated by the tequila and paper industry wastes in the western region of Mexico.

## 2. Materials and Methods

### 2.1. Materials

The ABF was donated by Tequila Jose Cuervo La Rojeña (Tequila, Jalisco, Mexico), and the CPB, also known as Kraft, was donated by a paper company in Tala, Jalisco, Mexico. The CC was provided by Asphalt Pavement and Construction Laboratories S.A. de C.V. (Ixtlahuacán de los Membrillos, Jalisco, Mexico). Sodium hydroxide (NaOH, 97% purity), sulfuric acid (H_2_SO_4_, 97% purity), potassium iodide (KI, 97% purity), and sodium chlorite (NaClO_2_, 97% purity) manufactured by Golden Bell Products Inc. (Orange, CA, USA). Anthraquinone (97% purity), cuprylenediamine (CED, 97% purity), sodium acetate (C_2_H_3_NaO_2_), and glacial acetic acid (CH_3_COOH) were purchased from Merck (Darmstdt, Germany). Potassium permanganate (KMnO_4_, 97% purity) was supplied by Monterrey Reactivo Analítico (Monterrey, N.L., Mexico). Sodium thiosulfate (Na_2_S_2_O_3_, 96% purity) was obtained from Karal Reactivos Analíticos (León, Gto., Mexico). Throughout all procedures, Milli-Q water with a resistivity of 18.2 MOhm × cm at 25 °C was used.

### 2.2. Obtaining Celluloses

#### 2.2.1. Cellulose from Agave Tequila Bagasse (ABP)

The procedure was adapted from the work done by Gallardo-Sánchez et al. [20] and was followed to obtain the ABP. First, the ABF was washed and de-medullated, then subjected to a full drying process at ambient temperature. Subsequently, 1 kg of dry, clean bagasse (ABF) was placed in a self-made batch steel reactor containing a solution of 20% NaOH and 0.1% anthraquinone (6:1 ratio) under a constant temperature of 170 °C for 90 min and a pressure of 7.73 kg/cm^2^ (110 psi) [20]. The cellulosic pulp obtained was washed with water to eliminate the residual liquor. The washed pulp was cleaned in a depurator (Lorentzen and Wettre, model F102, manufactured in Zurich, Switzerland) with a 0.40 mm grid sieve to remove impurities. Finally, the ABP cellulose was fluffed using a universal mill (IKA, model Mühle M20, manufactured in the Wilmington, NC, USA) to separate the pellets without disrupting the cellulose structure.

#### 2.2.2. Cellulose from Corrugated Paperboard (CPB)

The CPB consists of three paper layers: two liner faces (internal and external) and a central layer formed by B-flute corrugated paper. The cellulose used in this work was obtained based on the assumption that a pulping process had already been conducted for the paperboard’s manufacture. The corrugated paperboard was soaked in water until the pulp separated. It was then refined for 10 min using a Pila Valley refiner. Afterward, the pulp was air-dried at room temperature and finally processed in a universal mill (IKA, model Mühle M20, manufactured in the USA) to separate the fibers.

#### 2.2.3. Commercial Cellulose (CC)

The commercial cellulose fiber (CC) used in this study is distributed in Mexico for drainage and viscosity control in asphalt mixtures. It is made from waste paper products, has a gray appearance, and is available in the market either in pellet form or as separate fibers. In this work, the fiber was used in its separate form as received.

### 2.3. Chemical Properties of Celluloses

For the three types of cellulose (Agave tequila bagasse ABP, corrugated paperboard CPB, and commercial CC), the Kappa Number (KN), ash content, viscosity, degree of polymerization (DP) and elemental analysis were determined. The procedure for each determination is described below.

#### 2.3.1. Kappa Number (KN)

The KN was determined according to the standard TAPPI T236 om-06 2006 [33]. A higher KN value indicates a higher lignin content. Once the KN value is known, the percentage of lignin (also known as “Klason” lignin) can be obtained using the following equation:

(1) % lignin=(0.15)×(KN)

#### 2.3.2. Ash Content

The ash content provides an approximate estimate of the quantity of mineral salts and other inorganic materials present in the cellulose. Based on the standard TAPPI T211 om-85 [34], the ash content is obtained using the following Equation (2). In this case, the procedure was performed twice:

(2) % ash=(A×100)/B

where A is the weight of ash (g), and B is the weight of the ash sample without moisture (g).

#### 2.3.3. Viscosity (µ) and Degree of Polymerization (DP)

To evaluate the degradation that the cellulose molecular chain may have experienced due to the pulping and bleaching processes for each one of the used sources, the viscosity value (µ) was obtained according to the standard norm TAPPI T230 om-19 [35]. Therefore, four determinations were made per cellulose and the value was calculated using Equation (3):

(3) $$\mu =1.052\times \left(t_{p}\right)\times \left(C\right)$$

where t_p_ is the time (s) it takes for the solution to pass through the viscometer, and C is the constant of the capillary viscometer.

Using the relative viscosity (η_rel_) calculated with Equation (4), along with the data [η]c, the intrinsic viscosity (η) was obtained using Equation (5):

(4) $$\eta_{\mathrm{rel}}=t_{p}/t_{o}$$

(5) η=[η]c/c

where t_o_ is the time (s) it takes for the solution without a sample (blank) to pass through the viscometer, [η]c is the value obtained from Table 3 of the standard ASTM D1795 2013 [36], and c is the ratio between the weight of the sample (0.150 g) and the solvent (30 mL).

Through the relationship between viscosity (µ) and intrinsic viscosity (η), a simple expression for the degree of polymerization (DP) is obtained, as shown in expression (6):

(6) DP=(0.75×η)^(200/181)

#### 2.3.4. Elementary Analyses

The combustion elemental analysis technique can determine the total content of each element present in a sample, whether of organic or inorganic nature and in both solids and liquids. The equipment used for this analysis was a combustion elemental analyzer (Leco model TruSpec^®^ Micro, manufactured in the St. Joseph, MI, USA). In this analyzer, the elements are quantified relative to the initial weight of the sample, which is subjected to complete combustion at temperatures up to 970 °C. This article reports on the measurement of carbon, hydrogen, nitrogen, and sulfur using this equipment.

### 2.4. Morphology of Celluloses

The morphology of the celluloses was analyzed using scanning electron microscopy (SEM) with a high-resolution microscope (TESCAN MIRA 3, Micra Nano-technology, Mexico City, Mexico). The analysis was conducted at an accelerating voltage of 10 kV and a working distance of 15 mm. Before analysis, each sample was coated with a gold layer through sputtering for 30 s using the SPI-module sputter coater (Micra Nanotecnología, Mexico City, Mexico).

### 2.5. Spectroscopy of Celluloses

#### 2.5.1. Fourier Transform Infrared Spectroscopy (FTIR_ATR)

FTIR spectra were obtained for the three cellulose types using a spectrophotometer (Spectrum two; PerkinElmer, Mexico City, Mexico) employing the attenuated total reflectance (ATR) technique. The spectrum was constructed from an average of 36 scans over a frequency range of 4000 to 400 cm^−1^, and this procedure was performed in duplicate.

#### 2.5.2. X-Ray Diffraction (XRD)

X-ray diffraction (XRD) analysis was conducted using an X-ray diffractometer (Malvern Panalytical, model Empyream, Mexico City, Mexico) operating at 40 kV and 30 mA. The analysis was performed with an incidence angle ranging from 5° to 70°, a step size of 0.2°, and a duration of 30 s per step, utilizing CuKa radiation, a Ni filter, and a graphite monochromator.

### 2.6. Calorimetric Properties of Celluloses

Differential Scanning Calorimetry (DSC) and Thermogravimetric Analysis (TGA) were conducted on each cellulose sample using a TA Instruments DSC Discovery calorimeter (manufactured in the United States and provided by Waters S.A. de C.V., Ciudad de México, México). The DSC analysis involved heating the samples from −30 °C to 130 °C, followed by cooling and reheating from −30 °C to 400 °C at a rate of 10 °C/min. TGA measurements were performed using a TA Instruments Discovery TGA (also manufactured in the United States and provided by Waters S.A. de C.V.) across a temperature range of 50 °C to 600 °C with a heating ramp of 20 °C/min.

### 2.7. SMA Asphalt Mix

Schellenberg drainage tests (D) were performed on SMA asphalt mixtures using PG 64-16 asphalt with the addition of ABP, CPB, and CC cellulose, and each was tested independently. The AASHTO 325 standard [7] specifies that the percentage of commercial cellulose in an SMA mix should range from 0.3 to 0.5% by weight of the aggregates. Therefore, SMA asphalt mixtures were produced using various cellulose concentrations: 0.0, 0.1, 0.2, 0.25, 0.3, 0.4, 0.5, 0.7, and 1.0% by weight relative to the coarse aggregates.

Based on the SMA asphalt mix design with a nominal size of 9.5 mm, as developed for commercial cellulose by Magaña-Orozco [30], and meeting the permissible requirements established in the AASHTO 325 standard [7], the particle size distribution of the coarse and fine aggregates used in the SMA mix design was as follows: 15.5% 12.7 mm (1/2″) seal, 54.5% 9.5 mm (3/8″) seal, 25% sand, and 5.0% calcium carbonate. The optimum asphalt content (AC) was determined to be 8.0%. Based on this design, the grain size distribution and AC of the SMA mix were kept constant while the cellulose concentration was varied.

#### Schellenberg Drainage (D)

Asphalt drainage in the SMA mix was evaluated using the Schellenberg method, which is recommended for discontinuous mixes [37]. This method determines the amount of asphalt that drains due to an excess of asphalt in the mix, using Equation (7). The procedure involves heating the aggregates, asphalt, and cellulose to a temperature of 150 ± 5 °C. The mixing process is then carried out, and when the mixture reaches a weight of 1.0 kg, it is placed into a glass precipitate beaker and introduced into an oven at a constant temperature of 150 ± 5 °C for one hour. Finally, the material in the glass beaker is poured into a container, and the drained residue is weighed.

(7) $$D=\left(W_{3}-W_{1}\right)/\left(W_{2}-W_{1}\right)\cdot 100$$

where D is the material drainage in %, W_1_ is the weight of the glass beaker in g, W_2_ is the weight of the glass beaker plus the total material in g, and W_3_ is the weight of the glass beaker plus the retained material in g.

During the pouring of the material from the glass beaker into the container, no pressure should be applied. The material must simply fall freely. This procedure was followed for all SMA mixtures prepared with the different celluloses (ABP, CPB, and CC) at concentrations of 0.0, 0.1, 0.2, 0.25, 0.3, 0.4, 0.5, 0.7, and 1.0% by weight of the aggregates. Three determinations were conducted for each concentration.

## 3. Results and Discussion

### 3.1. Chemical Properties of Celluloses

During the alkaline cooking process of *Agave tequilana* Weber var. Azul (ABF), the yield of ABP cellulose was 38%, which is similar to the 40% yield reported by Gallardo-Sánchez et al. [20], who used a rotary reactor in their study. In the case of the mechanical purification process of cellulose from corrugated paperboard, a 100% yield was obtained.

**Figure 1:**
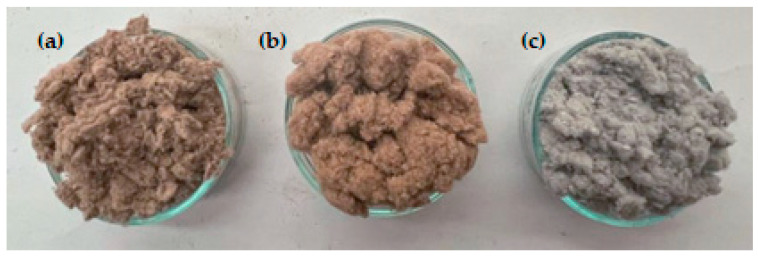
Analyzed celluloses: (**a**) ABP, (**b**) CPB, and (**c**) CC.

Figure 1 shows images of the studied cellulose type: (a) ABP in brown color, (b) CPB in a darker brown shade than that of ABP due to the amount of lignin present in the cellulose, and (c) commercial cellulose in gray color.

In addition to the color of cellulose, the KN serves as an indicator of the lignin content in pulp. Specifically, a higher KN value indicates a greater lignin concentration [33]. Alkaline cooking of *Agave tequilana* bagasse (ABF) resulted in delignification. Table 1 shows the chemical properties of each studied cellulose type. The KN value of ABF is 118.7 ± 0.7, indicating a lignin content of 17.8 ± 0.1%. These values are consistent with those reported by Pérez-Pimienta et al. [38], which range from 13 to 20 for *Agave tequilana* bagasse. During the cooking process, ABP cellulose was obtained from the ABF. This process produced ABP with a KN value of 13.9 ± 0.8, resulting in a lignin content of 2.1 ± 0.1%, evidencing a delignification of 88%. This indicates that ABP cellulose has a lower lignin content compared to the values reported by Gallardo-Sánchez et al. [20], who found a KN value of 23 ± 3 for ABP. Cellulose from corrugated paperboard (CPB) exhibited a KN value of 67.0 ± 0.4, corresponding to a lignin content of 10.1 ± 0.1%. In contrast, CC showed KN and percent lignin values of 30.9 ± 2.4 and 4.6 ± 0.4%, respectively.

The ash content corresponds to the remaining material calculated based on the dry weight of the sample after incineration. The TAPPI T211 om-85 [34] states that, in most cases, the ash content of paper and paperboard consists of inorganic chemical and mineral residues from pulp manufacturing, as well as metal materials from piping and machinery. Table 1 shows that ABP cellulose has the lowest ash content at 7.4 ± 0.9%, while CC cellulose presents the highest ash content (20.7 ± 0.1%) compared to the other celluloses. This higher content is due to the presence of inks, heavy metals, greases, and mineral fillers such as calcium carbonate, along with high starch content, which were not the focus of this study. In this context, Singh et al. [39] mention that the more cellulose is recycled, the more impurities it will contain, resulting in a higher ash content. Furthermore, Singh et al. [39] describe the ash content of corrugated paperboard as ranging from 1% to 10%, making the obtained value for CPB ash content (7.5 ± 0.2%) consistent with the literature.

ABF fiber presents a low value of 2.6 ± 1.5%, where Pérez-Pimienta et al. [38] report an ash content for *Agave tequilana* bagasse between 4% and 6%. Notably, ABP shows an ash content of 7.4 ± 0.9% (see Table 1). Despite both ABF and ABP originating from the same source, ABF grown in volcanic soils tends to accumulate silicate, potassium, and calcium crystals [40], which are retained in the ABF fiber and are difficult to remove during the pulping process. Therefore, after the delignification of ABP cellulose through the alkaline cooking process, the ratio of inorganic to organic matter increases, resulting in an increase in the ash content of ABP.

Table 1 shows the intrinsic viscosity (η) and the degree of polymerization (DP) of the celluloses used. CPB cellulose exhibits η and DP values of 505.78 ± 0.68 mL/g and 729.23 ± 1.1, respectively. These values are significantly higher compared to those of ABP and CC celluloses. In the second instance, ABP cellulose shows η = 482.72 ± 14.22 mL/g and DP = 692.60 ± 22.5. These values are lower than those reported by Gallardo-Sánchez et al. [20], which indicate an intrinsic viscosity of 830 mL/g and a degree of polymerization of 750 from a cellulose bleaching process. However, the DP value obtained from ABP (see Table 1) is similar to that reported by Ghosh et al. [41], where they studied rayon soluble-grade cellulose with a degree of polymerization of 678.27, which is associated with a high content of short fibers. The lowest η and DP values are presented by CC cellulose due to its origin from recycled paper, which has undergone multiple recycling processes. This has caused modifications in the molecular structure of the cellulose during pulping, resulting in values of η = 302.36 ± 46.85 mL/g and DP = 413.45 ± 70.7. Notably, as the value of η increases, the DP value of cellulose also increases, as described by Li et al. [42]. The DP indicates the number of repeating units (monomers) found in a polymer chain; CPB has a higher DP and contains longer, more flexible cellulose fibers compared to ABP and CC.

**Figure 2:**
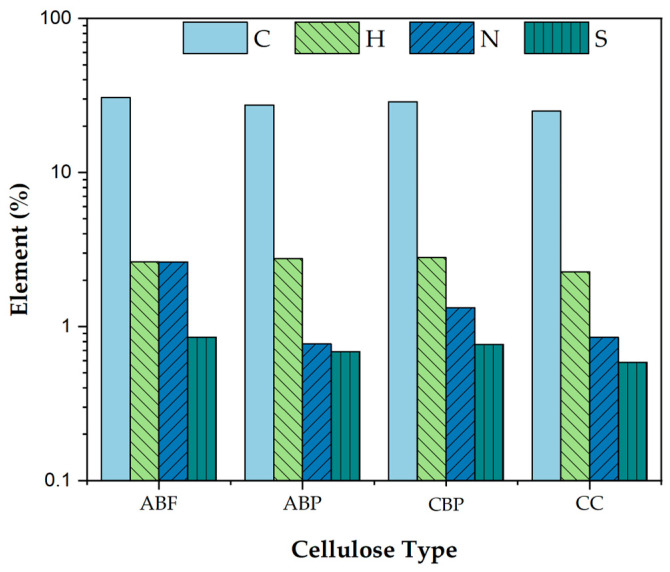
Elemental composition of Carbon (C), Hydrogen (H), Nitrogen (N) and Sulfur (S) in ABF and the studied celluloses: ABP, CPB, and CC.

On the other hand, Figure 2 shows the elemental analysis of each of the celluloses used. As they contain abundant cellulose and hemicellulose, a significant presence of hydrogen (H) and high carbon (C) content is observed. However, there are no significant changes in the C and H elements when comparing the ABF fiber with the ABP, CPB, and CC celluloses. The results of the elemental analysis show that the C values are 28.96 ± 2.35%, while the H values are 2.62 ± 0.24%. Regarding nitrogen (N), which is associated with the presence of lignin, the ABF presented an N content of 2.62%. When pulping is performed, accompanied by delignification to produce ABP cellulose, the N value drops to 0.77%, the lowest among the celluloses. This coincides with its lignin percentage of 2.1 ± 0.1%, which is also the lowest compared to the other celluloses. CPB cellulose, having the highest lignin content (% lignin = 10.1 ± 0.1), shows the highest N value at 1.33%. CC cellulose exhibits a lignin content of 4.6 ± 0.4% with a nitrogen content of 0.85%. Considering the sulfur oxide (SO) content found, the values are consistently below 1%, so this element is disregarded due to the potential measurement error of the equipment.

### 3.2. Morphology of Celluloses

**Figure 3:**
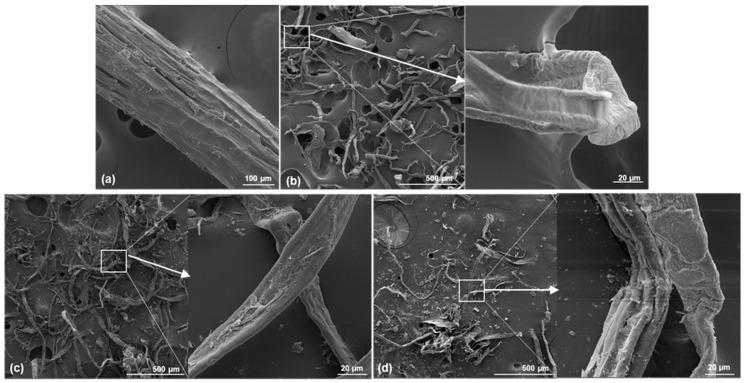
SEM micrographs from studied celluloses: (**a**) ABF, (**b**) ABP, (**c**) CPB, and (**d**) CC.

Figure 3a shows the micrograph of the transversal section of a long ABF fiber at a scale of 100 µm. The observed morphology is typical of bagasse fiber, formed by the union of several fibrils with an average diameter of approximately 254.71 ± 6.89 µm. The fibrils are packed together to form larger diameter fibrils, resembling an electric cable, with noticeable small spaces between them. This structure includes lignin, cellulose, amorphous waxes, and low molecular weight sugars, characteristic of bagasse from *Agave tequilana* Weber var. Azul. The fiber surface is not uniform; it has undulations along its length and small pores that facilitate water passage and retention, as shown by Gallardo-Sánchez [20].

In Figure 3b, observed at a scale of 500 µm, ABP cellulose shows separation of the fibrils due to the alkaline cooking process, which eliminated 88% of the lignin and hemicelluloses, as well as low molecular weight polysaccharides. ABP cellulose features thin fibrils with lengths similar to those of ABF but with considerably smaller diameters (a reduction of 86%), averaging 35.63 ± 10.78 µm. The fibrils exhibit a rough, corrugated, and undulating surface, which may enhance adhesion with asphalt, as described by Li et al. [42].

Figure 3c displays the morphology of CPB fibers at a scale of 500 µm. The lengths of these fibers are similar to those of ABF and ABP fibers; however, their diameters are around 18.44 ± 6.82 µm, representing reductions of 93% and 48% compared to ABF and ABP fibers, respectively. The morphology of commercial cellulose, shown in Figure 3d at a scale of 500 µm, presents diameters of approximately 29.26 ± 8.56 µm, closely resembling the shape of CPB cellulose. However, the fibril surface is much more corrugated and non-uniform, displaying folds and openings along the longitudinal axis. This condition is attributed to the high degree of recycling of CC cellulose, indicating significant structural damage consistent with its degree of polymerization (DP).

### 3.3. Spectroscopy of Celluloses

#### 3.3.1. Fourier Transform Infrared Spectroscopy (FTIR_ATR)

**Figure 4:**
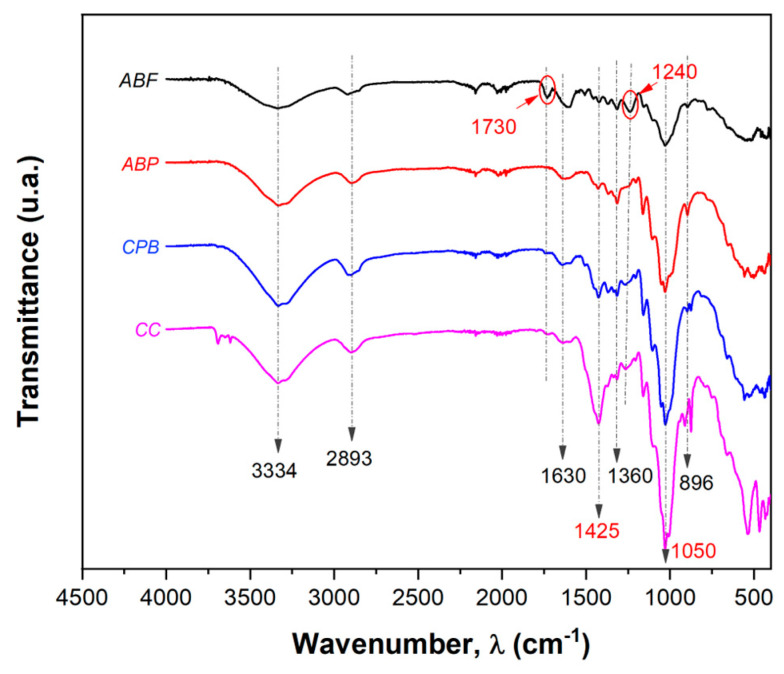
FTIR_ATR spectra of the ABF fiber and the ABP, CPB, and CC celluloses.

Cellulose fibers contain hydroxyl groups, methyl and side chains, aromatic groups, conjugated double bonds, and other active groups. From this, it can be deduced that there are several functional groups in both cellulose fibers and asphalt, indicating potential affinity between them, as described by Li et al. [32]. The spectra of the celluloses are presented in Figure 4, where it can be observed that the celluloses, regardless of their origin, display similar spectra. At a transmittance value of 3334 cm^−1^, there is a broad stretching band corresponding to the O-H groups present in ABF, ABP, CPB, and CC celluloses. This band contributes to the formation of hydrogen bonds in the structure of carbohydrates and cellulose [43,44]. The low-intensity peak at 2893 cm^−1^ present in all four celluloses corresponds to the stretching vibration of the C-H group, primarily due to the presence of cellulose and hemicellulose [20,32,43,45,46].

The absorption band at 1630 cm^−1^ present in ABP, CPB, and CC cellulose and at 1607 cm^−1^ in ABF fiber is attributed to the absorbed water in the lignocellulosic materials [20,44,45]. Even though the celluloses were subjected to a previous drying process, the FTIR tests provide evidence that they were not completely dry, as indicated by Ma et al. [45]. The band found at 1360 cm^−1^ is related to the bending vibrations of the C-H and C-O groups in polysaccharide aromatic rings [44], while a peak at 1030 cm^−1^ represents the C-O bond attributed to the polysaccharides present in cellulose [20,32,45].

At a transmittance of 1730 cm^−1^, the ABF fiber shows a stretching band of the carbonyl group (C=O), attributed to the presence of acetyl esters and carbonyl aldehydes in the hemicellulose and/or lignin [20,32,44]. At 1240 cm^−1^, a peak is associated with the out-of-plane stretching vibration of the C-O bond in the aryl group of lignin [44,45]. These two peaks present in ABF fiber have disappeared in ABP cellulose due to the delignification process but are observed with very low intensity in CPB and CC celluloses.

Regarding the crystallinity of celluloses, at a transmittance of 1050 cm^−1^, a C-O-C band corresponding to the vibration of the pyranose ring was identified. Additionally, a band at 896 cm^−1^ is characteristic of the β-glycosidic bond between the anhydroglucose units of cellulose [44]. Finally, at 1425 cm^−1^, the transmittance corresponds to the CH_2_-symmetric bending of cellulose [43], which is most intensely observed in CC and CPB celluloses.

Each peak area of the FTIR spectra shown in Figure 4 was obtained through deconvolution. Table 2 presents the area ratios, relating the area of each FTIR peak to the area of the peak at 896 cm^−1^, which is constant across all spectra, as reported by Gallardo-Sánchez et al. [20]. Analyzing the transmittance signals at 1730 and 1240 cm^−1^, which are associated with the presence of lignin and/or hemicelluloses, it is notable that the ABF fiber presents the highest value, indicating a greater presence of lignin. The ABP shows a lower value compared to ABF due to the delignification that occurs during alkaline cooking. In contrast, the highest area ratios among the celluloses are found in paperboard cellulose (CPB), while CC cellulose exhibits intermediate values. For the peaks at 1630, 2893, and 1050 cm^−1^, attributed to adsorbed water and C-H and C-O-C groups, respectively, the area ratios remain similar for ABP, CPB, and CC.

#### 3.3.2. X-Ray Diffraction (XRD)

**Figure 5:**
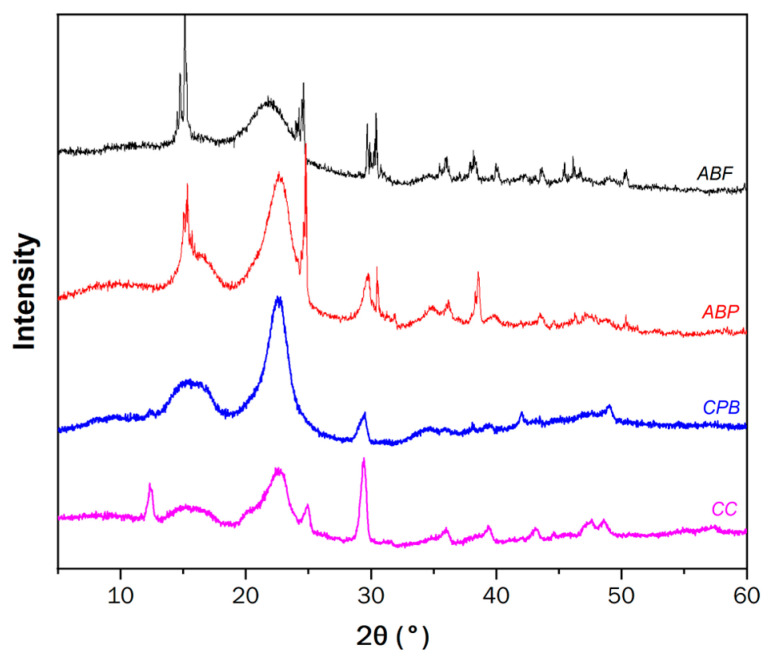
XRD diffractograms of ABF fiber and ABP, CPB, and CC celluloses.

The degree of crystallinity of the cellulose was determined using X-ray diffraction (XRD). The sharp peaks are associated with crystalline phases, while the broader peaks correspond to amorphous phases [20,28]. Figure 5 displays the diffractograms for ABF, ABP, CPB, and CC celluloses. Notably, there are signals associated with cellulose at varying magnitudes and amplifications. A small, unique peak at 12° in CC cellulose is related to type II cellulose, which has low crystallinity [47]. The peaks around 16° represent the amorphous components, such as low molecular weight hemicellulose, lignin, and wax compounds [33,37], identified as the (1 −1 0) plane [13,20,46]. These peaks exhibit higher magnitudes in CPB and CC celluloses. At approximately 23°, there is a peak representing the crystalline portion of cellulose [42], associated with the crystalline plane (2 0 0). This peak is more intense in CPB and ABP celluloses and less intense in CC cellulose, indicating its low crystallinity. Upon analyzing the ABF fiber and ABP cellulose, it is observed that this peak is sharper in ABP. This increase in the crystalline part (at 22.7°) is attributed to the partial removal of hemicelluloses and lignin during the alkaline cooking process. This observation is consistent with reports by Gallardo-Sánchez et al. [20] and Navarro-Hermosillo [48], who noted a peak in the crystalline band at 22° for nanocrystals and soluble-grade cellulose pulp derived from the bagasse of *Agave tequilana* Weber var. Azul.

Li et al. [42] mentioned that the crystallinity of a fiber is related to its thermal stability, absorption capacity, and mechanical properties. According to Equations (8) and (9), the crystallinity index (CI) and the percentage of crystallinity (%Cr) can be calculated as follows:

(8) $$\;\mathrm{CI}=\left(I_{22}-I_{\mathrm{am}}\right)/I_{22}$$

(9) $$\%\mathrm{Cr}=\left(\frac{I_{22}}{I_{22}+I_{\mathrm{am}}}\right)\cdot 100$$

where I_22_ is the maximum intensity of reflection in the crystalline planes of cellulose (22.7°, Figure 5), I_am_ is the maximum intensity of reflection in the amorphous planes (16° for CPB and ABP, and 2θ = 12° for CC cellulose, Figure 5).

Derived from this mathematical process, a crystallinity index (CI) of 0.44 and 0.23 was obtained for CPB and ABP cellulose, respectively, while a value of 0.17 was obtained for CC cellulose. Regarding %Cr, values of 64.2% and 56.4% were obtained for CPB and ABP cellulose, respectively, with 54.8% for CC cellulose. This indicates that CPB cellulose contains more than 60% crystalline composition, while ABP has 56.4% and CC has 54.8%. These percentages suggest that the molecules or atoms of cellulose are organized in an ordered and repetitive manner, demonstrating that CPB exhibits greater strength and hardness compared to ABP and CC. However, CC cellulose has less rigidity and resistance due to its pulping process. This observation is supported by the total crystallinity index (TCI) shown in Table 3, which is defined as the absorbance ratio obtained by relating the transmittances at 1360 cm^−1^ and 2893 cm^−1^ [49,50], as derived from the FTIR spectra. From Table 3, it is evident that the highest TCI is observed in CPB cellulose, followed by ABP, with the lowest value found in CC cellulose.

### 3.4. Calorimetric Properties of Celluloses

**Figure 6:**
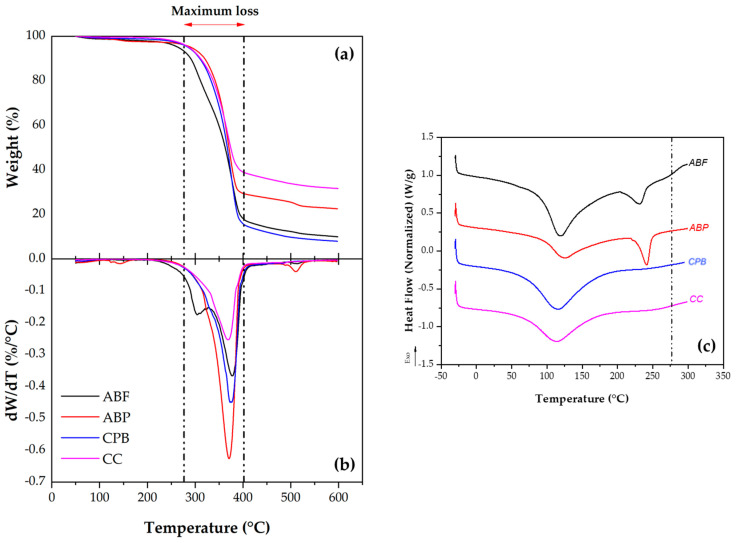
(**a**) Thermograms obtained from TGA, (**b**) TGA derivative, and (**c**) DSC of ABF fiber and ABP, CPB, and CC celluloses. The dotted lines delimit the temperature range where the samples lose the most significant weight.

The TGA analysis was performed to identify the thermal resistance behavior of the basic compounds in each cellulose, as these will be subjected to working temperatures of 150 ± 5 °C during the manufacture of SMA asphalt mixtures for flexible pavements. The TGA results are shown in Figure 6a, indicating the weight loss as the temperature increases in the studied celluloses. Remarkably, as shown in Figure 6a, over the temperature range of 50° to 190°, there is a small weight loss of about 2%, attributed to the evaporation of physically absorbed water [48,51,52,53]. Furthermore, at temperatures between 275 °C and 402 °C, ABP and CC celluloses experience material losses of 69% and 78%, respectively, while ABF and CPB celluloses lose a high percentage of material, at 90% and 92% in relation to their initial weight. It is evident that all celluloses suffer the greatest weight loss between 275 °C and 402 °C, which is mostly attributed to the presence of cellulose, hemicellulose, and other unstable materials [42,54]. Notably, ABP and CC demonstrate higher thermal degradation resistance throughout the entire temperature range tested.

Figure 6b shows the derivative of TGA weight loss against temperature. It is observed that the maximum degradation point occurs in CPB cellulose at a temperature of 374 °C. Similarly, the degradation point of ABP cellulose is at 370 °C, while for CC cellulose, it occurs at 368 °C, which is the lowest value. The degradation point as a function of temperature is associated with both the percentage of crystallinity (%Cr) and the degree of polymerization (DP). This indicates that CPB cellulose presents a superior crystalline arrangement (%Cr = 64.2%), as observed in the diffractograms, due to its high degree of polymerization (DP = 729.23). Therefore, this cellulose requires a higher temperature to degrade. In contrast, CC cellulose has the lowest degradation point at the lowest temperature (368 °C), attributed to its lower crystallinity (%Cr = 54.8%) and lower degree of polymerization (DP = 413.45).

Likewise, the thermograms obtained from DSC tests (see Figure 6c) show an endothermic peak observed in the ABF fiber and all the studied celluloses (ABP, CPB, and CC). It is notable that between 115 °C and 125 °C, there is a loss of water due to evaporation, as was observed in the TGA results. A second endothermic peak has been detected in ABF and ABP, in the range from 230 °C to 240 °C. This temperature is associated with the fusion process that may occur simultaneously with the degradation of the material, consistent with what was reported by Ma et al. [45] and Navarro-Hermosillo [48], who noted that cellulose fusions occur in the range of 227 °C to 360 °C. Therefore, cellulose degradation in ABF and ABP was observed in the DSC before the total degradation of cellulose was identified by TGA. Since the DSC thermograms were conducted up to 300 °C, the fusion of CPB and CC celluloses was not observed. Finally, based on the results obtained from TGA and DSC, it can be deduced that the ABF fiber and the ABP, CPB, and CC celluloses exhibit thermal stability at the working temperatures of asphalt mix production (150 ± 5 °C), as stated by Nikolaides [37].

### 3.5. Schellenberg Drainage (D)

**Figure 7:**
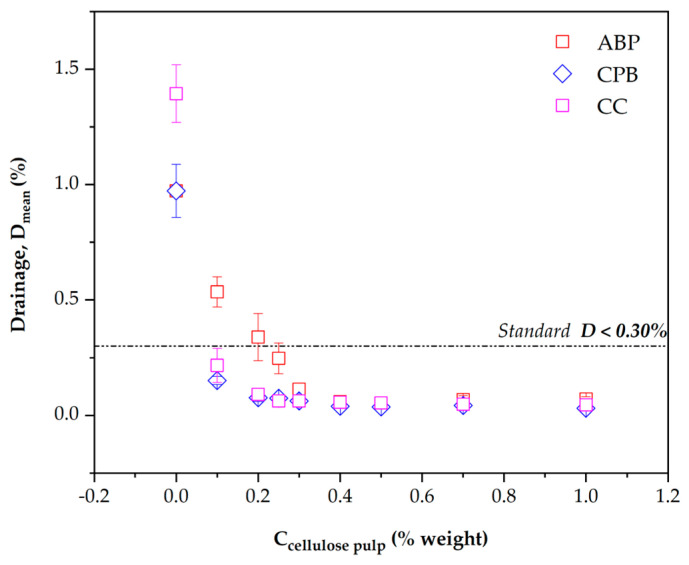
Schellenberg Drainage mean values (D_promedio_) as a function of cellulose concentration.

According to the AASHTO M325 standard [7], it is established that the drainage (D) in an SMA mix should not exceed 0.30%. Figure 7 shows the mean drainage value (D_mean_) of the SMA asphalt mixture fabricated using different concentrations of the studied celluloses (C_celulose_). The drainage values were obtained from three determinations performed for each concentration and type of cellulose.

From Figure 7, it is evident that CPB cellulose affects the SMA asphalt mixture from a cellulose concentration of 0.15%, as the mean drainage is less than the 0.30% established by the standard. Additionally, the AASHTO M325 [7] states that the use of commercial cellulose fiber (CC) should be in the range of 0.30% to 0.50%. Figure 7 shows that at 0.15% CC, the drainage is also less than 0.30%. Similarly, the use of ABP at 0.25% reduces the drainage of the SMA mixture below the 0.30% limit established by the standard. Based on the above, it is concluded that ABP and CPB demonstrate similar performance to CC cellulose, indicating that celluloses derived from agave and paperboard show effective stabilization behavior against drainage in the asphalt mixture.

**Figure 8:**
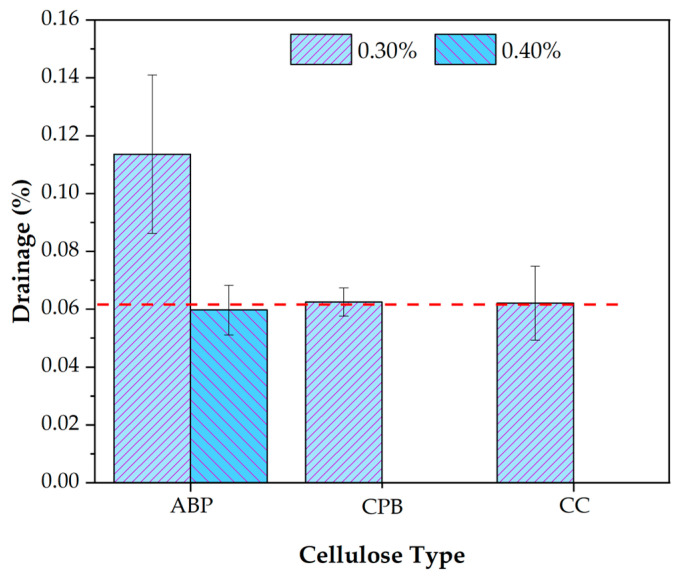
Schellenberg Drainage mean values (D_mean_) for ideal concentrations of 0.30% and 0.40% cellulose. The dotted line indicates the drainage value for CC.

Figure 8 shows the D_mean_ values at the cellulose concentration of 0.30% and 0.40% added to the SMA asphalt mixture. In the first instance, a D value of 0.062 ± 0.013% [11,55] is observed for the CC cellulose, which is taken as a reference since it is the commercially distributed cellulose. For a D value of 0.062 ± 0.005%, CPB cellulose closely approximates the behavior of CC cellulose, making it an excellent candidate to inhibit the drainage of the SMA mixture to the same magnitude as CC cellulose.

On the other hand, ABP cellulose is effective at a concentration of 0.30%, with a D value of 0.114 ± 0.027%. This value is higher than that of CC cellulose but still below the limit of 0.3% specified by the standard [7]. Therefore, at a concentration of 0.30%, ABP is a functional quantity for inhibiting drainage in the SMA mixture. However, to achieve the D value of CC cellulose, an additional 0.10% ABP concentration is necessary, resulting in an ideal concentration of 0.40% for ABP cellulose, which yields a D value of 0.059 ± 0.009%.

Finally, an analysis of variance (ANOVA) of the drainage value (D) was performed between the concentration and type of cellulose used (ABP, CPB and CC) using Origin, Version 2021. OriginLab Corporation, Northampton, MA, USA. For this study, nine concentrations were utilized: 0, 0.1, 0.2, 0.25, 0.3, 0.4, 0.5, 0.7, and 1% for each type of cellulose studied, with three repetitions per concentration. The ANOVA analysis was conducted at a 95% confidence level, revealing that both concentration and cellulose type were significantly different (*p* < 0.05), and that the interaction between concentration and cellulose type was also significant (*p* < 0.05).

The cellulose characteristics do not show significant changes in drainage for concentrations higher than 0.40% cellulose. However, at lower concentrations, the results for CC and CPB are similar, with ABP showing the most significant decrease in drainage. From the FTIR spectra, ABP has a lower lignin content (A_1240_/A_896_: 0.28 ± 0.04) compared to CC and CPB (A_1240_/A_896_: 0.75 ± 0.08 and 1.00 ± 0.07, respectively). This indicates that lignin content in the pulp is a determining factor in the drainage of asphalt mixtures. The A_2893_/A_896_ ratio also increases in the order of CC > CBP > ABP, while the A_1425_/A_896_ ratio decreases inversely. The peak at 2893 cm^−1^ can be attributed to the presence of celluloses and hemicelluloses; thus, drainage increases with hemicellulose content. Consequently, a higher concentration of ABP is required to inhibit drainage to the levels observed with CC or CBP. In contrast, the peak at 1425 cm^−1^ is mainly attributed to the crystalline structure of cellulose and varies according to the source [56]. This suggests that the source of cellulose plays an important role in its properties, influencing drainage. As shown in the results presented in Table 2, a higher A_1425_/A_896_ ratio correlates with better drainage efficiency. On the other hand, the total cellulose crystallinity index (TCI: A_1360_/A_2893_) and the degree of polymerization do not appear to be determining factors in drainage. However, they could directly influence the mechanical properties and performance of SMA asphalt mixtures.

From the above, it can be concluded that concentration and type of cellulose have a significant effect on the drainage response, demonstrating that ABP and CPB cellulose are viable alternatives to inhibit the drainage of the SMA mixture to values similar to those achieved by CC cellulose.

## 4. Conclusions

This paper focused on evaluating the influence of the chemical (lignin and ash content, viscosity, degree of polymerization, and elemental analysis), morphological (SEM), spectroscopic (FTIR-ATR and XRD), and calorimetric (ATG and DSC) properties of celluloses from *Agave tequilana* bagasse (ABP), corrugated paperboard (CPB), and commercial cellulose fiber (CC) as Schellenberg drainage (D) inhibitors of the SMA, in order to take advantage of these materials in the generation of value-added products and minimize the impact on the environment. The following conclusions are derived from this research:

- Limitations of CC cellulose: CC cellulose is primarily used in SMA mixtures as an asphalt drainage inhibitor and to control viscosity, but it does not significantly affect the mechanical behavior or performance of the mixture. Additionally, because this type of cellulose is not produced locally, its importation raises the final cost of the SMA mixture.
- Chemical Properties: The ash content and degree of polymerization were found to have no significant effect on the absorption of asphalt or in mitigating drainage in the asphalt mix. However, the Kappa Number, which is related to lignin content, and the nitrogen (N) content show a relationship. If the lignin content is very low, as observed in ABP cellulose, a higher concentration of cellulose is required to inhibit drainage to achieve the same effect as CC cellulose.
- Morphological Characteristics: Fiber absorption with asphalt reduces the drainage of the asphalt mixture. The micrographs of the three celluloses studied show that a greater number of undulations, a rough or corrugated texture, and varying filament diameters lead to increased absorption with asphalt. In contrast, CPB and CC celluloses exhibit more corrugation and smaller diameters, providing a greater contact surface between the cellulose and the asphalt, which improves absorption. The morphology of CPB and CC celluloses results from the recycling processes of paper and cardboard. Prolonged recycling leads to increased degradation of the cellulose, negatively affecting its morphology, with CC cellulose being the most damaged. In contrast, ABP cellulose is a virgin pulp, exhibiting a smoother fiber surface without undulations and featuring larger filament diameters. This morphology results in a lower impact on drainage due to reduced absorption between the cellulose and asphalt.
- Spectroscopic Analysis: Lignin removal in ABP is shown by faint bands at 1730 cm^−1^ (C=O) and 1240 cm^−1^ (C-O), which are less noticeable in CPB and CC. In addition, ABP has a lower lignin content than CC and CPB, indicating that lignin content in the pulp significantly affects the drainage of asphalt mixtures. Moreover, it is evident that the absorbance ratio increases in the order CC > CBP > ABP; conversely, this indicates a decrease in other ratios. This relates to the peak at 2893 cm^−1^, which is attributed to the presence of celluloses and hemicelluloses. As hemicellulose content increases, drainage also increases, necessitating a higher ABP concentration to inhibit drainage at levels comparable to CC or CBP. The peak at 1425 cm^−1^ primarily corresponds to the crystalline structure of cellulose and varies according to the source. This observation suggests that the source plays a crucial role in determining the properties of cellulose, thereby influencing the drainage performance of the SMA mixture.
- Crystallinity Assessment: The crystallinity obtained from X-ray diffraction demonstrates evidence of crystallinity among the three celluloses. CPB has a crystallinity index (CI = 0.44) and percentage of crystallinity (%Cr = 64.2%), indicating that more than 60% of its structure is crystalline, which contributes to its greater strength and toughness. ABP presents a CI of 0.23 and a %Cr of 56.4%, showing lower crystallinity compared to CPB. CC has the lowest values (CI = 0.17 and %Cr = 54.8%), suggesting low crystallinity, stiffness, and strength. This indicates that the pulping process affects the structural organization of cellulose, with CPB being the most crystalline and resistant. This observation is corroborated by the total crystallinity index (TCI) and is related to the degree of polymerization (DP), both of which are higher in CPB, followed by ABP, with CC cellulose exhibiting the lowest values. This results in improved mechanical and performance behavior of the SMA mixture when using CPB.
- Performance as Drainage Stabilizers: CPB and ABP are excellent alternatives to CC cellulose for drainage stabilization, with CPB being the most effective at low concentrations. This effectiveness is attributed to its morphology—including roughness, waviness, filament length, orientation, and diameter, as well as its lignin content, degree of crystallinity, and the type of cellulose source.

In conclusion, the production of value-added products derived from the residues of the tequila and cardboard industries, in the form of cellulose pulp, represents an alternative solution as a drainage inhibitor in SMA asphalt mixtures, with concentrations similar to those used with CC cellulose. This work demonstrates that cellulose source, lignin content, and morphology influence drainage. At the same time, crystallinity and degree of polymerization could contribute to the mechanical and performance behavior of the SMA mixture. Therefore, future research will evaluate the mechanical, performance, calorimetric, and morphological behavior of the SMA mixture at the optimal concentration of cellulose pulp, besides efforts to optimize the cellulose pulp extraction process to reduce impurities.

## Figures and Tables

**Table 1:** Chemical properties of ABF and cellulose: ABP, CPB and CC, respectively.

Cellulose Type	Kappa Number KN	% Lignin	% Ash	Intrinsic Viscosity η (mL/g)	Degree of Polymerization DP (Unitless)
ABF	118.17 ± 0.7	17.8 ± 0.1	2.6 ± 1.5	-	-
ABP	13.9 ± 0.8	2.1 ± 0.1	7.4 ± 0.9	482.72 ± 14.22	692.60 ± 22.5
CPB	67.0 ± 0.4	10.1 ± 0.1	7.5 ± 0.2	505.78 ± 0.68	729.23 ± 1.1
CC	30.9 ± 2.4	4.6 ± 0.4	20.7 ± 0.1	302.36 ± 46.85	413.45 ± 70.7

**Table 2:** Area ratio of FTIR spectra (A_λ_/A_896_).

Wavenumber (λ)	Area Ratio (A_λ_/A_λ896_)			
	ABF	ABP	CPB	CC
3334	9.50 ± 1.95	4.92 ± 0.23	4.50 ± 0.41	4.17 ± 0.39
2893	5.19 ± 0.24	2.82 ± 1.01	2.75 ± 0.85	1.73 ± 0.03
1730	0.87 ± 0.22	0.13 ± 0.08	0.49 ± 0.05	0.11 ± 0.03
1630	2.93 ± 0.39	0.67 ± 0.10	0.72 ± 0.06	0.33 ± 0.08
1425	1.12 ± 0.09	0.66 ± 0.09	1.46 ± 0.10	2.53 ± 0.13
1360	0.70 ± 0.07	1.41 ± 0.08	1.27 ± 0.05	0.70 ± 0.01
1240	2.66 ± 0.21	0.28 ± 0.04	1.00 ± 0.07	0.75 ± 0.08
1050	8.09 ± 0.56	6.67 ± 0.04	5.95 ± 0.03	7.58 ± 0.07

**Table 3:** Absorbance ratio from FTIR spectra (A_1360_/A_2893_), CI and %Cr from XRD.

	ABF	ABP	CPB	CC
TCI = A_1360/2893_	0.89 ± 0.03	0.80 ± 0.01	0.87 ± 0.07	0.72 ± 0.04
CI	-	0.23	0.44	0.17
%Cr	-	56.44	64.18	54.75

## Data Availability

The original contributions presented in the study are included in the article, further inquiries can be directed to the corresponding author.
